# Transporter gene acquisition and innovation in the evolution of Microsporidia intracellular parasites

**DOI:** 10.1038/s41467-018-03923-4

**Published:** 2018-04-27

**Authors:** P. Dean, K. M. Sendra, T. A. Williams, A. K. Watson, P. Major, S. Nakjang, E. Kozhevnikova, A. V. Goldberg, E. R. S. Kunji, R. P. Hirt, T. M. Embley

**Affiliations:** 1 Institute for Cell & Molecular Biosciences, Medical School, University of Newcastle upon Tyne, Framlington Place, Newcastle upon Tyne, NE2 4HH UK; 2 School of Biological Sciences, Life Sciences Building, University of Bristol, 24 Tyndall Avenue, Bristol, BS8 1TQ UK; 3 Bioinformatics Support Unit, William Leech Building, Framlington Place, Newcastle-upon-Tyne, NE2 4HH UK; 4 Medical Research Council Building, Mitochondrial Biology Unit, University of Cambridge, Cambridge Biomedical Campus, Hills Road, Cambridge, CB2 0XY UK

## Abstract

The acquisition of genes by horizontal transfer can impart entirely new biological functions and provide an important route to major evolutionary innovation. Here we have used ancient gene reconstruction and functional assays to investigate the impact of a single horizontally transferred nucleotide transporter into the common ancestor of the Microsporidia, a major radiation of intracellular parasites of animals and humans. We show that this transporter provided early microsporidians with the ability to steal host ATP and to become energy parasites. Gene duplication enabled the diversification of nucleotide transporter function to transport new substrates, including GTP and NAD^+^, and to evolve the proton-energized net import of nucleotides for nucleic acid biosynthesis, growth and replication. These innovations have allowed the loss of pathways for mitochondrial and cytosolic energy generation and nucleotide biosynthesis that are otherwise essential for free-living eukaryotes, resulting in the highly unusual and reduced cells and genomes of contemporary Microsporidia.

## Abstract (web summary)

Microsporidia are obligate intracellular parasites that infect both humans and animals. Here, Dean et al. perform ancient gene reconstruction and functional assays to investigate the evolution and functional diversification of nucleotide transporters which are key to the parasite's intracellular lifestyle.

## Introduction

Microsporidia are obligate intracellular parasites related to fungi that infect both immuno-competent and immuno-compromised humans^1^ and commercially important animals including fish, silkworms and honeybees^2^. Microsporidia can only complete their life cycle inside a host cell and survive in the external environment as resistant thick-walled spores^3^. The intracellular parasites grow rapidly and undergo several rounds of division before differentiating into new spores that are released into the environment to repeat the infection cycle. All microsporidian genomes have a highly reduced coding content comprising approximately 2000 to 3000 protein-coding genes and all species have lost the mitochondrial pathways for making ATP^4–6^, retaining a minimal genome-lacking mitochondrion^7^ (called a mitosome) only for its essential role in Fe/S protein biogenesis^8,9^. Although some species have retained genes for glycolysis, published data suggest that this pathway functions mainly in spores and is not used for making ATP in actively replicating parasites^6,10^. Other species, including the major human pathogen *Enterocytozoon bieneusi* have lost glycolysis altogether and thus have no independent means of making their own ATP^5,11^. This raises the question of how intracellular parasites acquire the enormous amounts of ATP and other nucleotides that they need to support their rapid growth and replication. It has been estimated that it takes at least 10^9^ ATP molecules just to make one *E. coli* cell^12^ and ATP demand by the larger and more complex cells of Microsporidia is likely to be much higher. The loss of indigenous pathways for energy generation means that intracellular parasites must now obtain all of this ATP from the infected host cell.

Genome analyses suggest that reduction in metabolic capabilities is the predominant mode of microsporidian genome evolution^4–6,9^ and that this has been supported by expansion of transporter gene families to compensate for pathway loss^13^. Given their predicted essential roles in supporting parasite growth and replication, surprisingly few of these transport proteins have been functionally characterised. Exceptions include the nucleotide transport (NTT) proteins that are expressed in the plasma membrane of *Encephalitozoon cuniculi* and *Trachipleistophora hominis* and which can transport ATP in heterologous transport assays using *E. coli*^14,15^. Phylogenetic analyses^16–18^ suggest that NTT transporters were acquired by horizontal gene transfer into the common ancestor of Microsporidia and *Rozella allomycis*, a fungal endoparasite^17,19^ belonging to the paraphyletic group Rozellomycota^19^ that also contains *Mitosporidium daphniae*^20^, and which is mainly known from environmental sequencing datasets^19^.

Horizontal gene transfers into eukaryotes have been suggested to be important drivers of adaptive evolution but experimental data supporting this hypothesis is limited^21^. To test the hypothesis that the horizontal acquisition of NTT transporters has played an important role in the adaptation of Microsporidia, we used phylogenetic methods and ancestral sequence reconstruction^22^ to infer the sequences of ancestral NTTs at two key points in their evolutionary history. Functional assays show that the reconstructed NTTs can transport ATP and hence would have provided early Microsporidia with the capacity to become intracellular energy parasites. To investigate how NTTs have evolved since their initial acquisition, we characterised the NTTs of three different contemporary Microsporidia species that can infect humans. The results show that NTT function has evolved to increase the range of purine nucleotides transported and to include a change in transport mechanism by individual NTTs to allow the net import of nucleotides for parasite growth and biosynthesis. The evolution of NTT function has enabled the loss of endogenous pathways for nucleotide biosynthesis and energy generation making Microsporidia dependent on NTT-mediated import for their survival. Our work demonstrates the fundamental importance of nucleotide transport proteins for a major group of medically and economically important obligate intracellular parasites infecting most animal groups.

## Results

### Ancestral sequence reconstruction of NTT transporters

**Fig. 1:**
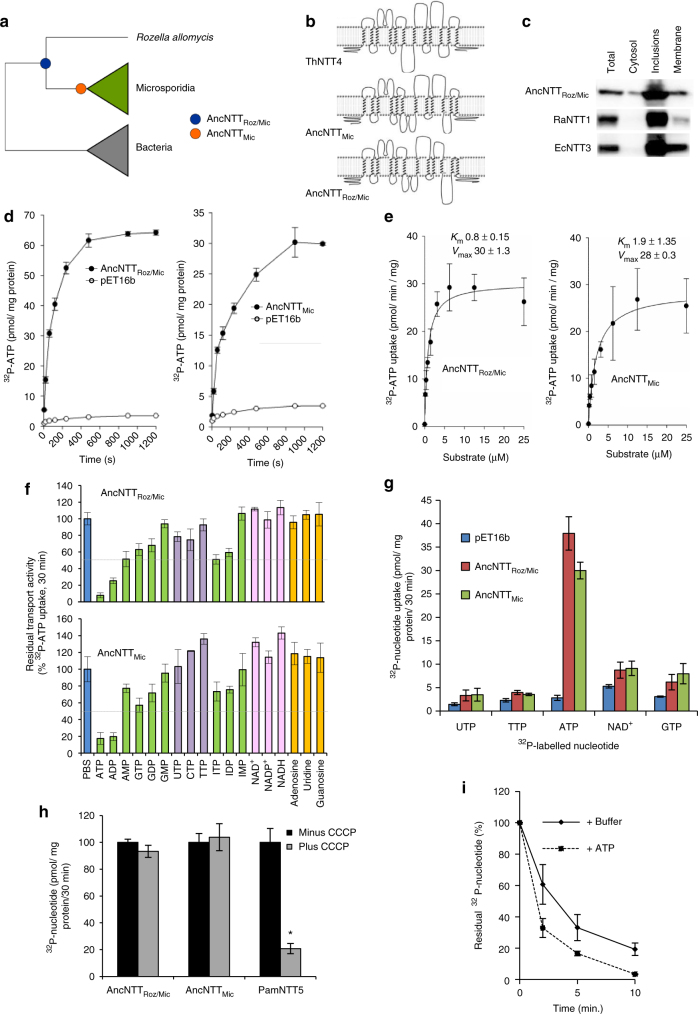
Ancestral reconstruction and functional characterisation of nucleotide transporters. **a** Schematic representation showing the position of the nodes in the NTT phylogenetic tree for which ancestral sequences (AncNTT_Roz/Mic_ and AncNTT_Mic_) were inferred and functionally characterised. We obtained point estimates of the ancestral NTT protein sequences by selecting the amino acid with the highest posterior probability at each site in the alignment (Supplementary Data 1 and 2). **b** Inferred secondary structure and membrane topology of one contemporary (ThNTT4) and the two ancestral sequences, predicted using HMMTOP. **c** Truncated western blot of fractionated *E. coli* expressing different NTTs detected using an anti-HIS antibody. Ancestral gene = AncNTT_Roz/Mic_, *E. cuniculi* *=* Ec, *R. allomycis* = Ra. Total = sonicated bacteria, Inclusions = 20,000 g pellet, Membranes = 150,000 g pellet, Cytosol = supernatant after 150,000 × *g* spin. Complete blots are shown in Supplementary Figure 4. **d** Kinetics of [^32^P]-ATP uptake by ancestral NTTs expressed in *E. coli*; pET16b = empty vector control. **e** Substrate saturation curve for the uptake of [^32^P]-ATP in the presence of increasing concentrations of unlabelled ATP. Data is fitted to a Michaelis–Menten equation to determine *K*_m_ (µM) and *V*_max_ (pmol/min/mg) by iteration. **f** Competitive substrate inhibition against [^32^P]-ATP uptake. Competitors were at 50,000× excess over the radio-labelled ATP. Data points represent residual radioactivity within the bacteria after subtraction of the empty vector control. **g** Nucleotide uptake of [^32^P]-labelled pyrimidine (dTTP and UTP) or purine (ATP and GTP) nucleotides or NAD^+^ by the ancestral NTTs. **h** Effect of the protonophore CCCP on [^32^P]-nucleotide uptake by the two ancestral proteins and PamNTT5 of *Protochlamydia*. Significant difference (*) to the control was only seen for PamNTT5 (*p* < 0.05; one-way ANOVA). **i** Back-exchange assay whereby [^32^P]-ATP-loaded *E. coli* expressing AncNTT_Roz/Mic_ were incubated in the presence or absence (=Buffer) of unlabelled ATP. Data shows residual intracellular label in harvested *E. coli* cells. All data points represent means ± SD of at least three independent experiments

**Fig. 2:**
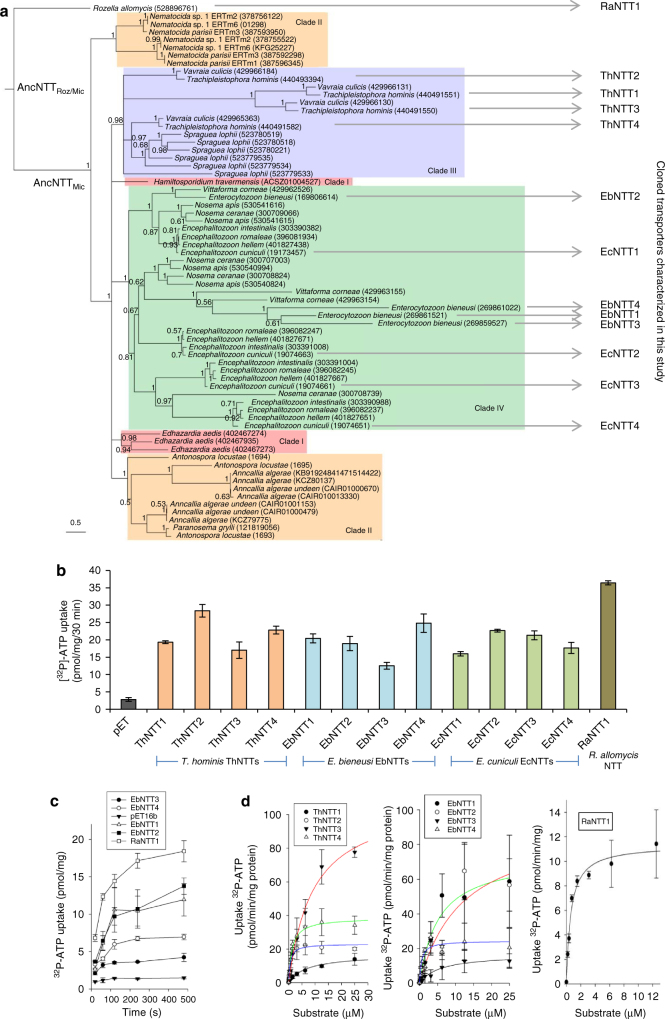
Phylogeny and ATP transport by Microsporidia and *Rozella* NTTs. **a** NTT phylogeny for the Microsporidia/*R. allomycis* clade of endoparasitic fungi inferred under the CAT+GTR model in PhyloBayes. The tree is the posterior consensus tree inferred under the CAT+GTR model, in which all relationships with posterior support <0.5 were collapsed. Branch lengths are proportional to the expected number of substitutions per site. Scale bar = 0.5 changes per site. A single-ancestral acquisition of a bacterial NTT gene is inferred in the common ancestor of Microsporidia and *Rozella* followed by independent gene duplications and family expansion during the radiation of Microsporidia. The NTTs that were functionally characterised in this study are indicated, including the two ancestral NTTs shown at the base of the tree. Support values are Bayesian posterior probabilities. **b** Uptake of [^32^P]-ATP by *E. coli* cells expressing NTTs for 30 min. All NTT gene and species names are given. ATP uptakes for all NTTs were significantly different (*p* < 0.05, one-way ANOVA) to the pET16b control. **c** Kinetics of [^32^P]-ATP uptake in *E. coli* expressing NTTs from *E. bieneusi* (EbNTT1–4) or *R. allomycis* (RaNTT1). **d** Substrate saturation curves for [^32^P]-ATP to determine *K*_m_ and *V*_max_ for NTTs from *T. hominis* (ThNTT1–4), *E. bieneusi* (EbNTT1–4), and *R. allomycis* (RaNTT1). Curves were fitted to the Michaelis–Menten equation and the *K*_m_ and *V*_max_ were calculated by iteration. All data points represent means ± SD of at least three independent experiments

The best fitting CAT+GTR model^23^ was used to infer a phylogeny for NTT sequences from Microsporidia, *Rozella allomycis*, and outgroup Bacteria. CAT+GTR is particularly appropriate for analysis of NTTs because it explicitly models the site-specific biochemical properties of the amino acid alignment, such as the preference for hydrophobic residues in transmembrane domains. Both the monophyly of all Microsporidia NTTs, and a sister-group relationship between Microsporidia and *Rozella*, were recovered with maximal posterior support (PP = 1) in the consensus tree (Figs. 1a and 2a). We used the *ancestral* program in the PhyloBayes package^23^ to sample the most probable amino acid at each site (see Supplementary Data 1 and 2) in the ancestral sequences corresponding to the common ancestors of Microsporidia (AncNTT_Mic_) and Microsporidia and *Rozella* (AncNTT_Roz/Mic_) (Fig. 1a**)**. Both inferred ancestral sequences contain residues known to be functionally important for NTTs (Supplementary Fig. 1 & 2)^24^ and HHPRED analyses demonstrate that, like contemporary NTTs, they are members of the Major Facilitator Superfamily (MFS; Supplementary Table 2). Analyses of the inferred secondary structure of the two ancestral protein sequences using HMMTOP^25^ suggested that they contain the necessary transmembrane domains in the correct orientation to fold and insert into membranes as a member of the MFS and NTT protein family (Fig. 1b**;** Supplementary Fig. 3a & 4). To test empirically whether the predicted ancestral proteins can insert into biological membranes and undertake the conformational changes that MFS transporters must adopt to transport substrates^26^, we used gene synthesis to recreate the ancestral proteins in the laboratory.

The ancestral sequences AncNTT_Roz/Mic_ and AncNTT_Mic_ were synthesised as codon-optimised genes for expression in *E. coli*, a heterologous host that can be used for transport assays with radio-labelled [α^32^P]-ATP^14,15^. Consistent with their predicted secondary structure (Supplementary Fig. 3a), the expressed proteins were detected in the *E. coli* membrane fraction (Fig. 1c; Supplementary Fig. 4). Both proteins also transported [α^32^P]-ATP in a time-dependent manner (Fig. 1d) and at high affinity as demonstrated by the low apparent *K*_m_ values (Fig. 1e; Supplementary Fig. 3b). Competition assays^14,27^ using a range of substrates to compete with [α^32^P]-ATP uptake indicated that the “ancestral” NTTs have relatively narrow substrate ranges, as only ATP and ADP consistently inhibited transport below 50% compared to the controls (Fig. 1f). Competition assays can only give an indication of whether a particular substrate might be transported because they do not actually measure transport of the cold substrate^14,27^, so we tested transport of radio-labelled UTP, dTTP, GTP and NAD^+^ by AncNTT_Roz/Mic_ and AncNTT_Mic_. Both of the ancestral NTTs exhibited strong uptake of radio-labelled ATP; at least a tenfold increase compared to the empty vector controls (Fig. 1g) and only weak uptake of the other tested nucleotides. Some members of the MFS transporter family, including some NTTs used by intracellular bacteria^27^, are symporters that can use proton gradients to drive net import^26^. The protonophore carbonyl cyanide m-chlorophenyl hydrazone (CCCP) can be used to dissipate the proton gradient^27^ to identify symporters experimentally. We therefore tested transport of radio-labelled ATP by both ancestral transporters in the presence or absence of CCCP, and included PamNTT5 of *Protochlamydia amoebophila*, an intracellular bacterial symbiont of *Acanthamoeba*, as a known symporter and positive control^27^. The addition of CCCP (Fig. 1h) did not inhibit transport indicating that AncNTT_Roz/Mic_ and AncNTT_Mic_ are ATP/ADP exchangers, a result that was supported by a counter-exchange assay for AncNTT_Roz/Mic_ (Fig. 1i). In these assays, [α^32^P]-ATP-loaded bacteria were exposed to buffer or buffer containing unlabelled external ATP, and the residual radiolabel associated with harvested bacteria followed over time. AncNTT_Roz/Mic_ exhibited a higher rate of export of radionucleotide when incubated with ATP (Fig. 1i), as reported previously for the *E. cuniculi* exchanger EcNTT3^14^. Together these data suggest that acquisition of an NTT gene by the common ancestor of Microsporidia and *Rozella* would have provided a tool to steal host ATP and thus to become an energy parasite.

### Functional analysis of NTTs from contemporary Microsporidia

To investigate the role(s) that NTT transporters play in contemporary parasite biology, we characterised the NTTs of three phylogenetically distinct Microsporidia (Fig. 2) that can infect humans as well as animals. The presence of NTT genes in all microsporidian genomes and the expansion of NTT gene copy number by independent lineage- and clade-specific gene duplication events (Fig. 2), coupled with the central roles of nucleotides in cellular metabolism, suggest that NTT-mediated transport plays an important role in the biology of contemporary Microsporidia. In classical theory, gene duplication is thought to provide an important pathway to evolutionary innovation because it can free individual gene copies to evolve new functions^28^. Expansion of transporter functional repertoire must have occurred during Microsporidia evolution because, in addition to the absence of pathways for ATP production, the early ATP-expensive steps needed for the de novo biosynthesis of the nucleotides needed to make DNA, RNA and nicotinamide cofactors are also missing from sequenced genomes^13,29^. To test the hypothesis that NTT transporter function has evolved after duplication, and thus enabled pathway loss, we investigated the functional biology of NTTs from *Encephalitozoon cuniculi* and *Trachipleistophora hominis* which cause opportunistic infections of humans^3^ and are among the few model species that can be grown in co-culture with mammalian cells. The NTTs from *Enterocytozoon bieneusi*, the most common cause of human infection and which cannot yet be grown in the laboratory^30^ were also characterised as was the single NTT of *Rozella allomycis*, the endoparasitic fungal sister lineage to Microsporidia. *Rozella* has less reduced gene content than Microsporidia including components of a mitochondrial electron transport chain for making ATP, and a lifestyle that alternates between a free-living motile zoospore and a naked endoparasitic stage^17^.

The draft genome sequence^5^ of *Enterocytozoon bieneusi* was used to guide synthesis of its four NTTs (EbNTT1–4) as codon-optimised genes for expression in *E. coli*. The four NTT genes from *E. cuniculi* (EcNTT1–4)^14^ and from *T. hominis* (ThNTT1–4)^15^ as well as the single NTT gene (RaNTT1) from *Rozella allomycis*^17^ were all cloned and expressed in *E. coli*. All of the NTTs transported [α^32^P]-ATP (Fig. 2b) in a time-dependent manner (Fig. 2c)^14,15^. To calculate an apparent *K*_m_ and *V*_max_ for the *T. hominis*, *E. bieneusi*, and *R. allomycis* NTTs, [α^32^P]-ATP uptake was performed in the presence of increasing concentrations of non-radio-labelled ATP (Fig. 2d). The low *K*_m_ values for all of these transporters are similar to published values for *E. cuniculi* NTTs^14^ (Fig. 2d; Supplementary Fig. 3b) and suggest that they have high affinity for ATP. Moreover, the *K*_m_ values for the Microsporidia and *Rozella* NTTs are well below the estimated ATP concentration for the cytoplasm of eukaryotes (~3 mM)^31^, suggesting that transport of ATP from infected host cells would not be limiting during the parasite life cycle.

**Fig. 3:**
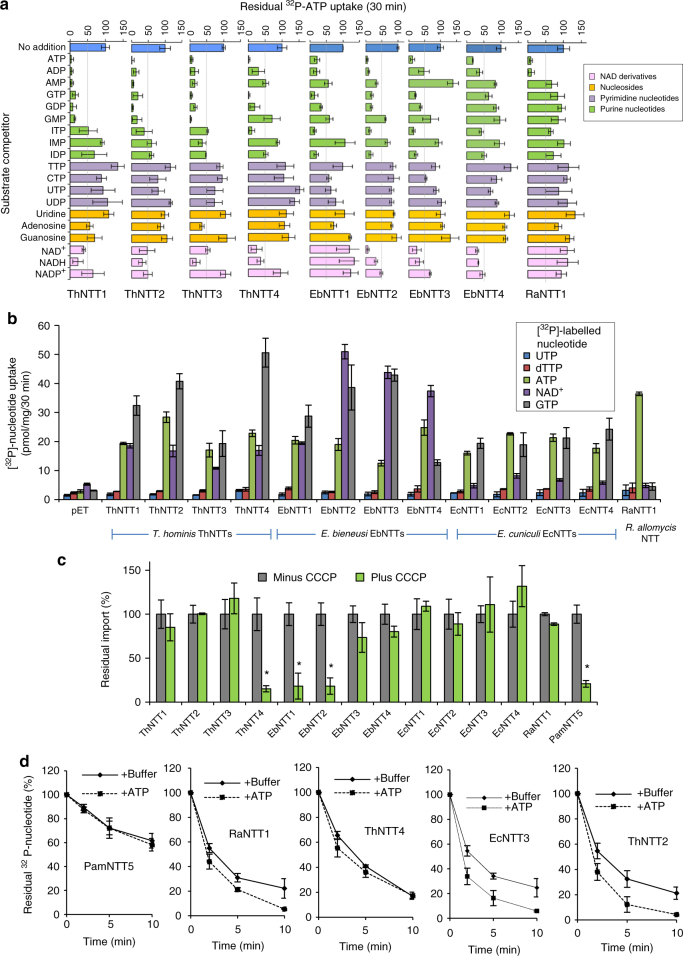
Substrate competition assays and nucleotide uptake assays for Microsporidia and *Rozella* NTTs. **a** Substrate competition assays whereby [^32^P]-ATP uptake by NTT-expressing *E. coli* was performed in the presence of 50,000 × excess unlabelled substrate. Th* T. hominis*, Eb* E. bieneusi,* Ra* R. allomycis*. **b** [^32^P]-nucleotide uptake assays with *E. coli* cells expressing NTT genes. Uptake of NAD^+^ by *E. cuniculi* NTTs was not significantly different to empty vector controls (*p* > 0.05, one-way ANOVA). Coloured bars show transport data for the indicated pyrimidine nucleotides (dTTP or UTP), purine nucleotides (ATP or GTP) or NAD^+^. pET empty vector control. **c** [^32^P]-nucleotide uptake by NTT-expressing *E. coli* in the absence (set to 100% after control was subtracted) or presence of carbonyl cyanide m-chlorophenyl hydrazone (CCCP). Significant difference at *p* < 0.05 (one-way ANOVA) is shown with *. **d** Back-exchange assay with [^32^P]-ATP-loaded *E. coli* incubated in the presence or absence of 100 μM unlabelled ATP. Data shows the residual radioactivity following washing of the bacteria. PamNTT5 is a positive control symporter from the bacterium *Protochlamydia amoebophila*^27^. All data (mean ± SD) is representative of at least three independent experiments

Analysis of the *E. bieneusi, E. cuniculi*, *T. hominis*, and *Rozella allomycis* genomes demonstrate that they lack genes for the enzymes needed to make purine and pyrimidine nucleotides de novo (Supplementary Fig. 5 and 6)^29^. To investigate if the Microsporidia NTTs can transport other nucleotides to fill these gaps, we carried out competition assays for NTT-mediated ^32^P-ATP uptake in the presence of an excess of a variety of individual cold purine and pyrimidine nucleotides, nucleosides, and nicotinamide derivatives. The results of the competition experiments and published data for *E. cuniculi*^14^, suggest that the NTTs of all three Microsporidia are purine nucleotide transporters with a preference for adenosine and guanosine triphosphates and diphosphates (Fig. 3a). By contrast, transport by *R. allomycis* RaNTT1 was strongly reduced only by cold ATP and ADP (Fig. 3a). The addition of cold ITP-reduced and IDP-reduced ATP transport below 50% by some Microsporidia NTTs (Fig. 3a), but these are unlikely to be substrates for nucleotide biosynthesis since the genomes of all three species lack the enzymes^29^ for incorporating ITP or IDP into cellular metabolism (Supplementary Fig. 6). ATP transport by some *T. hominis*, *E. cuniculi*^14^ and *E. bieneusi* NTTs was also inhibited to varying degrees by nicotinamide derivatives (mainly NAD^+^, NADH and NADP^+^) suggesting that these might be transported (Fig. 3a), as demonstrated previously for one of the NTTs (PamNTT4) of *Protochlamydia amoebophila*^32^. NADH and NADPH are predicted to be essential co-factors in core metabolic reactions in Microsporidia^13^ but genome analyses suggest that unlike *Rozella allomycis*, the Microsporidia species *E. cuniculi*, *T. hominis* and *E. bieneusi* cannot make NAD^+^ derivatives de novo (Supplementary Fig. 7). Retention of the NAD^+^ kinase and phosphohydrolase enzymes needed to interconvert between NAD^+^ and NADP^+^ (Supplementary Fig. 7) does suggest, however, that if NAD^+^ can be transported, then all three species could make NADH, NADP^+^ and NADPH.

To test the hypotheses of substrate transport generated by the competition data, we investigated the transport of radio-labelled UTP, dTTP, GTP and NAD^+^ by the individual NTTs when expressed in *E. coli*. The results of these experiments demonstrate that GTP is transported at high levels for all of the Microsporidia NTTs (Fig. 3b). This suggests that the two main classes of purine nucleotides are transported by Microsporidia NTTs and this is consistent with genome analyses that suggest that both types would need to be imported^13,29^. By contrast, we found no evidence for GTP transport above background by the single NTT of *R. allomycis* (RaNTT1), and thus it appears that transport by RaNTT1 is restricted to ATP and ADP among the substrates tested (Figs. 2b and 3). Radio-labelled ^32^P-NAD^+^ uptake assays in *E. coli* revealed that all of the NTTs from *T. hominis* and *E. bieneusi* could transport NAD^+^ (Fig. 3b). The weak NAD^+^ transport detected for two (EcNTT2 and EcNTT3) of the four *E. cuniculi* NTTs (Fig. 3b) was only slightly higher than, and not significantly different (*p* > 0.05) to, the empty vector control. The observation that the *R. allomycis* RaNTT1 did not transport NAD^+^ (Fig. 3b) is consistent with genome analysis that suggests that, unlike Microsporidia, *R. allomycis* can make this important cofactor (Supplementary Fig. 7).

The competition data provided no evidence that ATP transport by Microsporidia NTTs was significantly inhibited by pyrimidine substrates (Fig. 3a), despite evidence from genome analysis (Supplementary Fig. 5) that Microsporidia lack the enzymes needed to make pyrimidines de novo. Genome analyses do suggest that, if UTP were to be transported, then Microsporidia have the enzymes to make the other pyrimidine nucleotides needed for nucleic acid biosynthesis (Supplementary Fig. 5)^15,29^. We therefore investigated if radio-labelled UTP or dTTP were transported in our assays, but detected no convincing evidence for transport of either substrate by the Microsporidia or *Rozella* NTTs (Fig. 3). It has previously been suggested^33^ that a conserved family of Microsporidia putative transporters related to the *E. coli* NupG nucleoside transporter^13^ might be used by the parasites to import host cell nucleosides, potentially providing starting substrates for pyrimidine nucleotide biosynthesis. However, in addition to the absence of any published experimental data to support nucleoside transport by these parasite proteins, the genome of *E. cuniculi*^15^ and other Microsporidia^29^ appear to lack the kinases needed to use nucleosides for pyrimidine nucleotide biosynthesis^29^. This suggests that nucleoside import would not solve the pyrimidine deficit for these species.

**Fig. 4:**
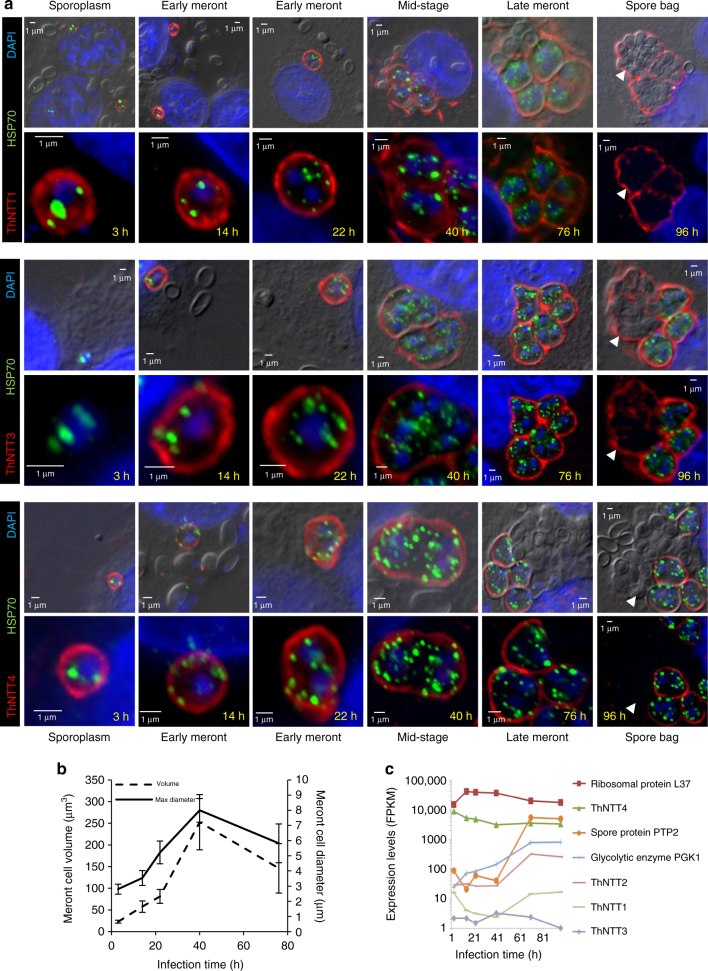
Protein localisation and transcript abundance for *T. hominis* NTT transporters over a time course of *T. hominis* infection. **a** Immunofluorescence time course (3–96 h) of rabbit kidney (RK-13) cells infected with *T. hominis* spores using published^15^ individual rabbit anti-ThNTT antibodies (red). Rat anti-HSP70^15^ (green) was used to label the mitosomes of intracellular parasites (meronts). The first time point at 3 h is shortly after injection of the *T. hominis* sporoplasm into the host cell when labelling by antisera to ThNTT1 and ThNTT4 is already apparent. The top DIC image shows the spore bags (arrows) at 96 h with superimposed labelling (red) by antisera to ThNTT1 and ThNTT3 but not by antisera to ThNTT4. Scale bar is 1 µm. **b** Increase in parasite biomass during *T. hominis* infection time course measured using cell diameter and cell volume. Diameter of parasite cells (minimum 100 counted) was taken at their widest point, and cell volumes were calculated using Axiovision software. Error bars are standard deviation. *N* = 3. **c** RNAseq analysis showing transcript abundance (log_10_ FPKM (Fragments per kilobase per million mapped reads)) for the four ThNTTs, spore protein (PTP2), a glycolytic enzyme (PGK1) and ribosomal protein L37 throughout the infection time course

**Fig. 5:**
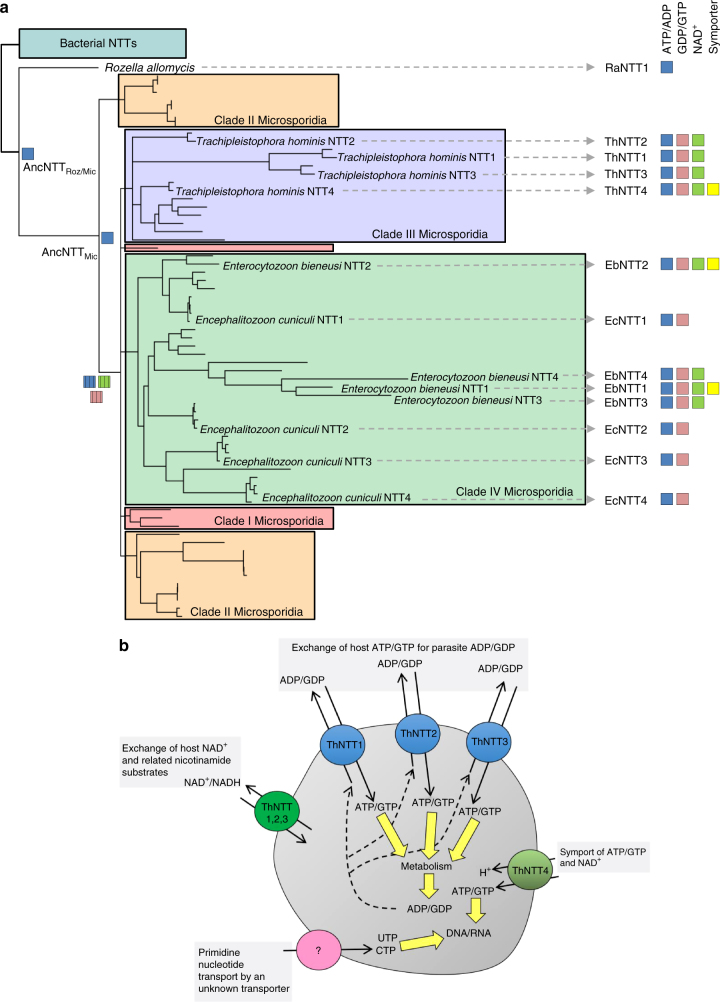
Gene duplication and evolution of Microsporidia NTTs. **a** Phylogeny of Microsporidia and *Rozella* NTTs and their transported substrates. ATP transport by the predicted ancestral NTTs AncNTT_Roz/Mic_ and AncNTT_Mic_ are shown at the respective nodes. The tree topology suggests that the common ancestor of Microsporidia clade III and IV could already transport both purine nucleotides and potentially NAD^+^ as indicated by the cross-hatched boxes. **b** A model for *T. hominis* NTT-mediated acquisition of energy and nucleotides from infected cells. NTTs are located at the parasite plasma membrane and can act as exchangers (ThNTT1-3) or symporters (ThNTT4) enabling energy parasitism or net nucleotide uptake, respectively. The transporters and/or pathways used by *T. hominis* to acquire pyrimidine nucleotides are currently unknown

Our data suggest that NTT substrate range has evolved over time facilitated by gene duplication, allowing the loss of parasite pathways for ATP and nucleotide biosynthesis. However, the inferred ancestral NTT phenotype of nucleotide exchange cannot provide the net import of nucleotides needed for nucleic acid synthesis and the increase in parasite biomass observed during intracellular infection (Fig. 4a,b). We therefore tested transport of radio-labelled ATP by all of the NTTs in the presence or absence of CCCP^27^. The results suggest that whereas the *Rozella* NTT and most of the Microsporidia NTTs have retained the ancestral exchanger phenotype (Fig. 3c), *T. hominis* ThNTT4, and *E. bieneusi* EbNTT1 and EbNTT2 have independently evolved into symporters capable of carrying out net nucleotide import (Figs. 3c and  5a). To investigate further, we carried out counter-exchange assays^14,27^ for a representative sample of both types of NTT (Fig. 3d). ThNTT4 and PamNTT5 exhibited no increased rate of export of radio-labelled nucleotide consistent with them being symporters^14,27^, while EcNTT3^14^, ThNTT2 and RaNTT1 exhibited a higher rate of export when incubated with ATP (Fig. 3d), suggesting that they are exchangers. Taken together, our data suggest that, in addition to using NTTs for energy parasitism, *E. bieneusi* and *T. hominis*, but not *E. cuniculi*, can use some of their NTTs for net import of NAD^+^ and purine nucleotides.

### Accelerated evolution and functionality of paralogous NTTs

Data from yeast^34^ has demonstrated that evolution following gene duplication can also include accelerated rates of sequence evolution and changes in gene expression or protein localisation. The differences in branch lengths (Fig. 2) observed for individual Microsporidia NTT paralogues relative to the *Rozella* outgroup is suggestive of gene-specific accelerated evolution and altered selective constraints. Indeed, a Bayesian relative rates test indicated that the 95% credible intervals for all of the Microsporidia NTT branch lengths do not overlap with that of the *Rozella* lineage, providing evidence of significant increases and differences in evolutionary rates after the split between Microsporidia and *Rozella* (Fig. 2; Supplementary Table 3). To investigate if these accelerated evolutionary rates are associated with changes in *Trachipleistophora hominis* NTT RNA transcript levels or protein localisation, we used published^15^ NTT-specific antibodies against ThNTT1, 3 and 4, coupled with RNAseq to investigate protein and RNA transcript abundance in a synchronised culture of *T. hominis* infecting Rabbit kidney cells (Fig. 4a). Apart from a single early time point for ThNTT3 (Fig. 4a), ThNTT transporters are present in the plasma membrane^15^ of intracellular vegetative cells to meet the enormous demand for host ATP and GTP imposed by parasite growth and replication (Fig. 4a,b). The very high levels of transcripts for parasite ribosomal proteins (exemplified by ribosomal protein L37 in Fig. 4c) needed for ribosome biogenesis^35^, provide further evidence of the severe burden imposed by the parasites on host resources. Interestingly, no mitosomal location was detected for any *T. hominis* NTT throughout the parasite life cycle, in contrast to the situation in *E. cuniculi* where one NTT (EcNTT3) is targeted to its mitosomes^14^. The functional data obtained in the present study and our previous work^14,15^, suggest that *T. hominis* has adopted a different nucleotide acquisition strategy (Fig. 5b) to *E. cuniculi* during their independent evolution.

The *T. hominis* NTT paralogues with the longest branch lengths in the tree are ThNTT1 and 3 (Fig. 2a), and these two genes also have the lowest abundance of RNA transcripts throughout the parasite life cycle (Fig. 4c). Intriguingly, antibody data suggest that ThNTT1 and 3 have undergone functional specialisation because they are the only two NTTs that associate with the sporophorous vesicle that surrounds maturing *T. hominis* spores (Fig. 4a). RNA transcript levels for ThNTT2 and ThNTT3 (but not ThNTT1) rise during spore formation and follow the increases observed for transcripts of polar tube protein 2 and PGK that are enriched in spores^6^. This suggests that ThNTT2 and 3 may have a particular role in supplying ATP for the parasites during spore formation. Transcript abundance for the symporter ThNTT4 is in the top 10% of expressed genes throughout the parasite life cycle and is between 100-fold and 1000-fold higher than any other ThNTT (Fig. 4c). In the case of ThNTT4 (see Fig. 5b), the functional shift from ancestral exchanger to symporter has been coupled with increased gene expression, providing critical complementary adaptations that can support rapid parasite growth.

## Discussion

Ancestral sequence recreation (ASR) brings with it a number of uncertainties associated with the reliability of ancient phylogenetic reconstructions^22^, but it nevertheless represents a powerful tool to test hypotheses about the function of genes in the deep past. Here, we provide experimental evidence that the inferred ancestral NTT sequences at two early and critical points in Microsporidia evolution (Fig. 1a), encode proteins that can insert into biological membranes and undergo the necessary conformational changes^26^ required to transport ATP and ADP. Moreover, the transport kinetics (apparent *K*_m_ and *V*_max_) of the ancestral proteins fall within the variation reported for contemporary NTTs^14,15,27^. Our results suggest that the acquisition of an NTT gene by the common ancestor of Microsporidia and *Rozella*, an organism that evolved after the *Rozella* lineage split from other fungi around 900 million years ago^36^, would have allowed the adoption of energy parasitism. We also show that NTT copy number and function (Fig. 5a) has evolved across the Microsporidia tree during the ensuing millennia, allowing Microsporidia to further adapt to their intracellular lifestyle and to lose endogenous pathways for nucleotide biosynthesis and energy generation. These losses mean that contemporary Microsporidia can only survive in the external environment as highly resistant spores^3^. Their success as intracellular parasites is attested by the very large number (>1000) of Microsporidia species described to date and their apparent ubiquity as parasites among animal groups^3^. The latter suggests that the enormous diversity of Microsporidia is linked to the explosive radiation of their animal hosts over the past 600 to 700 million years^37^. Genome analyses suggest that expansion of different transporter families through gene duplication is a general feature of the otherwise highly reduced Microsporidia proteome^13^, providing the tools to harvest the sugars, lipids, amino acids and other building blocks, that they no longer make for themselves. The huge metabolic drain on host resources imposed by the transporters of rapidly growing and replicating parasites is likely a major reason for the reduced host fitness and increased mortality observed in susceptible eukaryotic populations^1,2^.

## Methods

### Host cell culture and parasite propagation

The Microsporidia *Trachipleistophora hominis* (ATCC—PRA-404) was initially isolated from a human HIV/AIDS patient^38,39^ and is now routinely sub-cultured within the rabbit kidney cell line RK-13 (ATCC—CCL-37) at 33 °C in Dulbecco’s Modified Eagle Medium (DMEM), containing 10% FCS, penicillin (100 µg/ml) and streptomycin (100 µg/ml). Spores used in time course experiments were harvested from infected RK-13 cells grown in 20 175 cm^2^ tissue culture flasks. Cells were washed and scraped into phosphate buffered saline (PBS, pH 7.4) and then lysed by sonication. The released spores were purified on a Percoll gradient prior to adding them to uninfected RK-13 cells as reported previously^40^.

### Synchronising infection of RK-13 cells by *T. hominis* and RNASeq analysis

To synchronise the infection of *T. hominis*, healthy RK-13 cells grown in 150 cm^2^ round tissue culture dishes, were incubated with freshly prepared spores as previously described^40^ and then subjected to extensive washing after 2 h. Two dishes of cells were used for RNA purification^40^ at 3, 14, 22, 40, 70 and 96 h post-inoculation. Sequencing, including library preparation, was carried out using the Illumina stranded mRNA sample preparation kit, with sequencing carried out on two lanes of an Illumina HISeq 2500, producing a total of 27.5 M paired-end reads representing *T. hominis* transcripts. Sequencing reads were trimmed using CutAdapt to remove adaptor sequences and low quality 3′ sequence regions (set at the q -20 threshold). Trimmed reads were mapped to the *T. hominis* genome using TopHat2^41^ and transcripts were assembled and quantified using Cufflinks and CuffDiff as previously described^40^.

### Immunofluorescence microscopy of NTT transporters

Samples of infected RK-13 cells used in the RNASeq analysis were grown in parallel on 13 mm coverslips for immunofluorescence. At selected times post-infection, cells were washed twice in PBS prior to being fixed at −20C for 1–2 h with methanol/acetone (50:50). Fixed cells were washed in PBS, blocked in 1% milk in PBS and then labelled using published^15^ rabbit antisera (1:100) against *T. hominis* NTT transporters ThNTT1–4 or rat antisera (1:100) to *T. hominis* mitosomal Hsp70 (ThHSP70) which labels parasite-specific mitosomes, as previously described^15^. Cells were also stained with DAPI (Molecular Probes) and mounted in Vectashield Hard Set (VectorLabs). Microscopy was performed with a Zeiss AxioImager II epifluorescence microscope using a ×63 objective lens and images were processed using Axiovision software.

### Cloning and gene synthesis of nucleotide transporters

Primers for the amplification of NTT transporter genes are given in Supplementary Table 1. *Enterocytozoon bieneusi* genomic DNA was obtained from purified spores (kindly provided by Dr. Elizabeth Didier, Tulane University, USA) and NTT genes for sequence checking were PCR amplified and named EbNTT1–4. The synthetic NTT genes used for transport assays were codon-optimised for expression in *E. coli* and synthesised by GeneArt. The single NTT gene from *Rozella allomycis*^17^ was PCR-amplified from *R. allomycis* genomic DNA (kindly provided by Dr. Timothy James, University of Michigan, USA) and named RaNTT1. All transporter genes were cloned into pET16b (Novagen) in frame with an N-terminal deca-histidine tag and confirmed by sequencing. pET16b plasmids encoding *T. hominis* or *E. cuniculi* NTT transporters were prepared previously^14,15^.

### NTT expression, western blotting and bacterial fractionation

For all NTTs, expression was performed from a pET16b vector freshly transformed into *E. coli* Rosetta2 DE3 pLysS (EMDMillipore). Luria Broth was inoculated with individual colonies and shaken overnight at 37 °C prior to the inoculation of Terrific Broth (TB; Sigma). At OD_600_ 0.4–0.6, cells were chilled to 18 °C prior to the addition of 1 mM IPTG to induce gene expression for 16–18 h. Bacteria were chilled on ice, sedimented at 6000 × *g* for 5 min and washed twice with cold PBS. Standard western blot analysis was performed on 20 μg total bacteria lysate using an anti-polyHis antibody (H1029, Sigma) and the colonies expressing the highest levels of NTTs were used for subsequent studies. To assess membrane localisation, bacteria were sonicated followed by differential ultra-centrifugation to isolate a high-speed membrane fraction (20,000 × *g* × 10 min and 150,000 × *g* × 2 h) for western blot analysis.

### Nucleotide uptake assays

Prior to uptake experiments, freshly transformed colonies were screened for NTT expression by western blot (2–3 days prior) and colonies with the highest NTT expression levels used in subsequent experiments. For all uptake assays, bacteria were resuspended to an OD_600_ of 5.0 in PBS. Uptake assays, including competition assays, counter-exchange and dose response experiments were performed as previously described^14^ with minor modifications. Single endpoint experiments were performed for 30 min for all radio-labelled nucleotides. An empty pET16b vector was used as a control in all experiments. To determine the initial rate of transport, [^32^P]-ATP uptake activity was recorded in the presence of increasing concentrations of non-radio-labelled substrates as described previously^14^. Data was fitted with the Michaelis–Menten equation to determine the apparent *K*_m_ and *V*_max_ of transport. Back-exchange assays were carried out as described previously^14^, whereby *E. coli* cells were loaded for 30 min with [^32^P]-ATP, washed and incubated in PBS containing 100 μM cold ATP and the residual radioactivity was detected by filtering the bacteria through a 0.45 μm cellulose nitrate membrane filter at different times post-washing. Carbonyl cyanide m-chlorophenyl hydrazone (5 min; 250 μM CCCP; Sigma Aldrich) was used to dissipate the proton gradient prior to uptake assays. All radiochemicals were obtained from Hartmann except [^32^P]-nicotinamide adenine dinucleotide (NAD^+^), which was obtained from Perkin Elmer. Unlabelled nucleotides that were used in competition and uptake assays were obtained from Sigma Aldrich and prepared according to the manufacturer’s instructions. All uptake experiments were performed as independent triplicates and where indicated, significant difference was assessed using a one-way ANOVA performed using the statistical program SPSS.

### Phylogenetic analysis and inference of ancestral NTT sequences

We augmented the sequence sampling of a published NTT phylogeny^15^ with the NTT protein sequences from recently sequenced Microsporidia genomes using BLASTP searches at NCBI, using *T. hominis* ThNTT4 as the query. Sequences were aligned using MUSCLE^42^ and the alignment was manually edited to remove insertions found in single sequences and the very C-terminal end of the alignment, where there was no recognisable homology between the bacterial and eukaryotic sequences. The alignment can be downloaded from Figshare (10.6084/m9.figshare.5170729.v1) and is also included as a high-resolution pdf for on-screen visualisation (Supplementary Fig. 2**)**. We tested the fit of the single-matrix LG model^43^, and the site-heterogeneous CAT+Poisson and CAT+GTR^44^ models to the alignment using PhyloBayes 3.3^23^, in all cases using a discretised gamma distribution (four categories) to model among-site rate variation. Posterior predictive simulations indicated that only the CAT+GTR model provided an adequate fit to the alignment with respect to site-specific sequence composition, which is known to be an important factor in accurate phylogenetic inference^45^. We therefore used the CAT+GTR model both to infer the tree and to reconstruct ancestral sequences, using the ancestral program that is part of the PhyloBayes package. Figure 2 depicts the posterior consensus tree inferred under the CAT+GTR model, in which all relationships with posterior support <0.5 were collapsed. Branch lengths are proportional to the expected number of substitutions per site. We obtained point estimates of the ancestral NTT protein sequences in the Microsporidia common ancestor and in the common ancestor of Microsporidia and *Rozella* by selecting the amino acid with the highest posterior probability at each site in the alignment (Supplementary Data 1 and 2). Analyses of the secondary structure of the two inferred sequences was performed with HMMTOP^25^. Graphical representations of transmembrane topology were performed with TMRPres2D (http://bioinformatics.biol.uoa.gr/TMRPres2D). The inferred ancestral sequences have been submitted to Genbank (AncNTT_Roz/Mic_: MF279273; AncNTT_Mic_: MF279274).

To test whether the differences in branch lengths among paralogous Microsporidia NTTs observed in the Bayesian consensus tree were significant, we used a Bayesian relative rates test. We constructed 95% credible intervals for the patristic distances (that is, the summed branch length differences) from the common ancestor of Microsporidia and *Rozella* to the tips represented by each NTT that was functionally characterised in this study. These distances were computed on each tree sampled during the MCMC run, after discarding the first 50% of points as burn-in, and are summarised in Supplementary Table 3. The means and credible intervals for Microsporidia NTTs are clearly distinct from each other and from the shorter branch leading to *Rozella*, providing evidence for changes in evolutionary rate following gene duplication during the radiation of Microsporidia.

### Identification of nucleotide metabolic genes in Microsporidia

Microsporidia homologues were identified as described in refs. ^13,15,29^. In brief, Microsporidia protein families were constructed using Markov Clustering with all-against-all sequence similarity scores generated from PHMMER. Putative functions of Microsporidia proteins were inferred using COG annotation of the human or yeast homologue represented in the same cluster. As Microsporidia sequences are often highly divergent, HHsearch^46^ was also used to annotate Microsporidia clusters for which no COG hit was obtained. Homologues from *Rozella allomycis* were identified using analysis results from KAAS in addition to KEGG annotation available from the JGI Genome portal (http://genome.jgi.doe.gov). KASS was set to search against the default eukaryote representative sets with the addition of *Nosema ceranae, Neurospora crassa, Aspergillus oryzae, Sclerotina sclerotiorum, Phaeosphaeria nodorum, Tuber melanosporum* and *Cryptococcus neoformans*. The bi-directional best-hit method was used to assign homologues. To further identify potential homologues from Microsporidia and *Rozella*, manual curation using domain identification and Psi-blast and tblastn searches were also performed (https://blast.ncbi.nim.nih.gov/Blast.cgi).

### Data availability

Sequence data that support the findings of this study have been deposited in Genbank with the primary accession codes MF279273 and MF279274, and in Figshare.

## Electronic supplementary material

Supplementary Information

Description of Additional Supplementary Files

Supplementary Data 1

Supplementary Data 2
