# Supplementary material for: Transporter gene acquisition and innovation in the evolution of Microsporidia intracellular parasites
Source: Nat Commun. 2018 Apr 27;9:1709. doi: 10.1038/s41467-018-03923-4 (PMC5923384; doi:10.1038/s41467-018-03923-4)
Supplement: Supplementary file 1 — Supplementary Information [file 41467_2018_3923_MOESM1_ESM.pdf]

Supporting Information

Dean et al. **Transporter gene acquisition and innovation in the evolution of Microsporidia intracellular parasites**

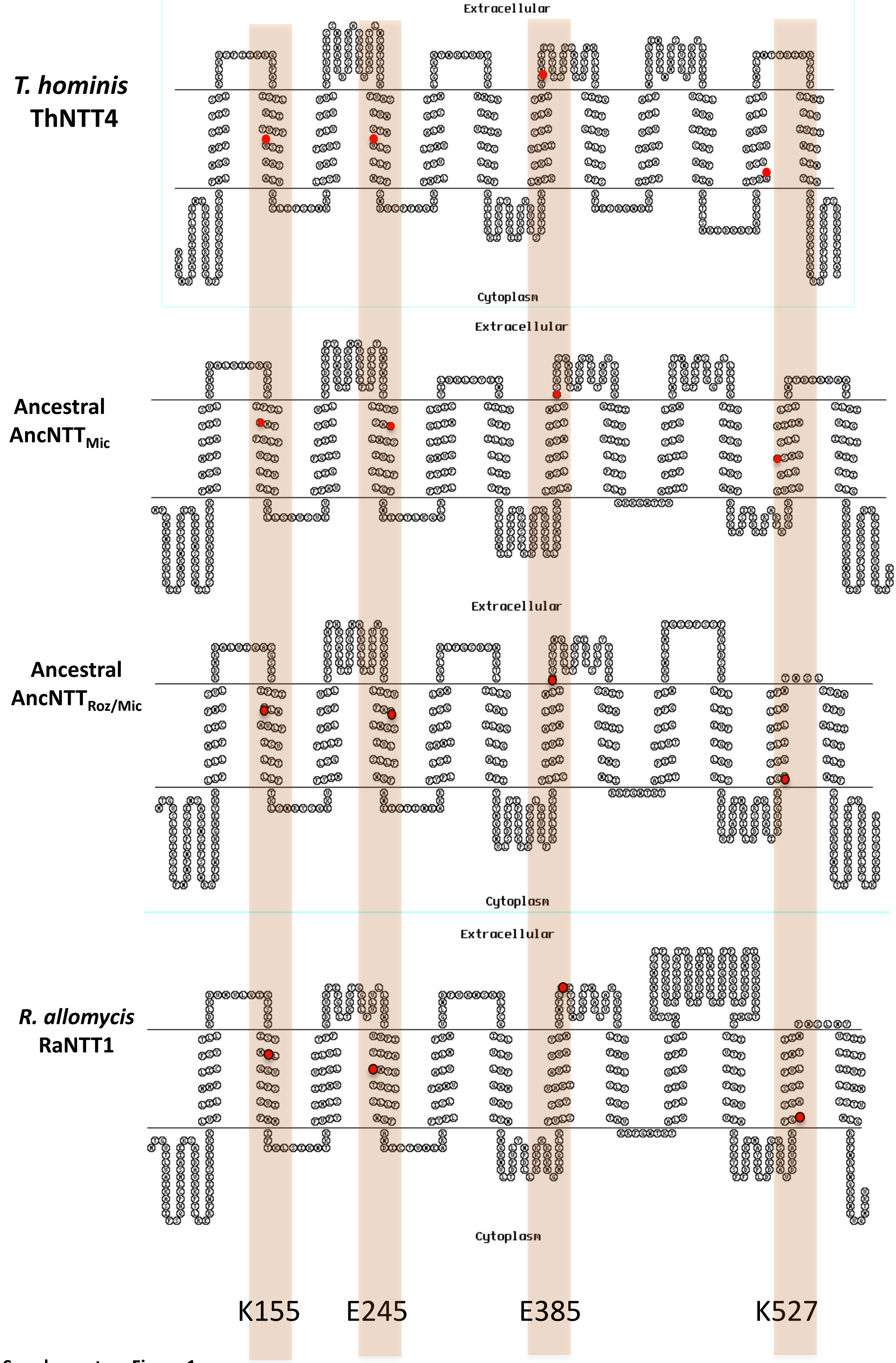

Supplementary Figure 1

Transmembrane representation of NTTs showing the critical residues (red) that are conserved in bacterial, plant and Microsporidia NTTs and also retained in the two ancestral sequences used in this study. The position of the indicated residues refers to those functionally characterised in *Arabidopsis thaliana* AATP1[24]. Graphical representations of transmembrane topology were performed with TMRPres2D (http://bioinformatics.biol.uoa.gr/TMRPres2D).

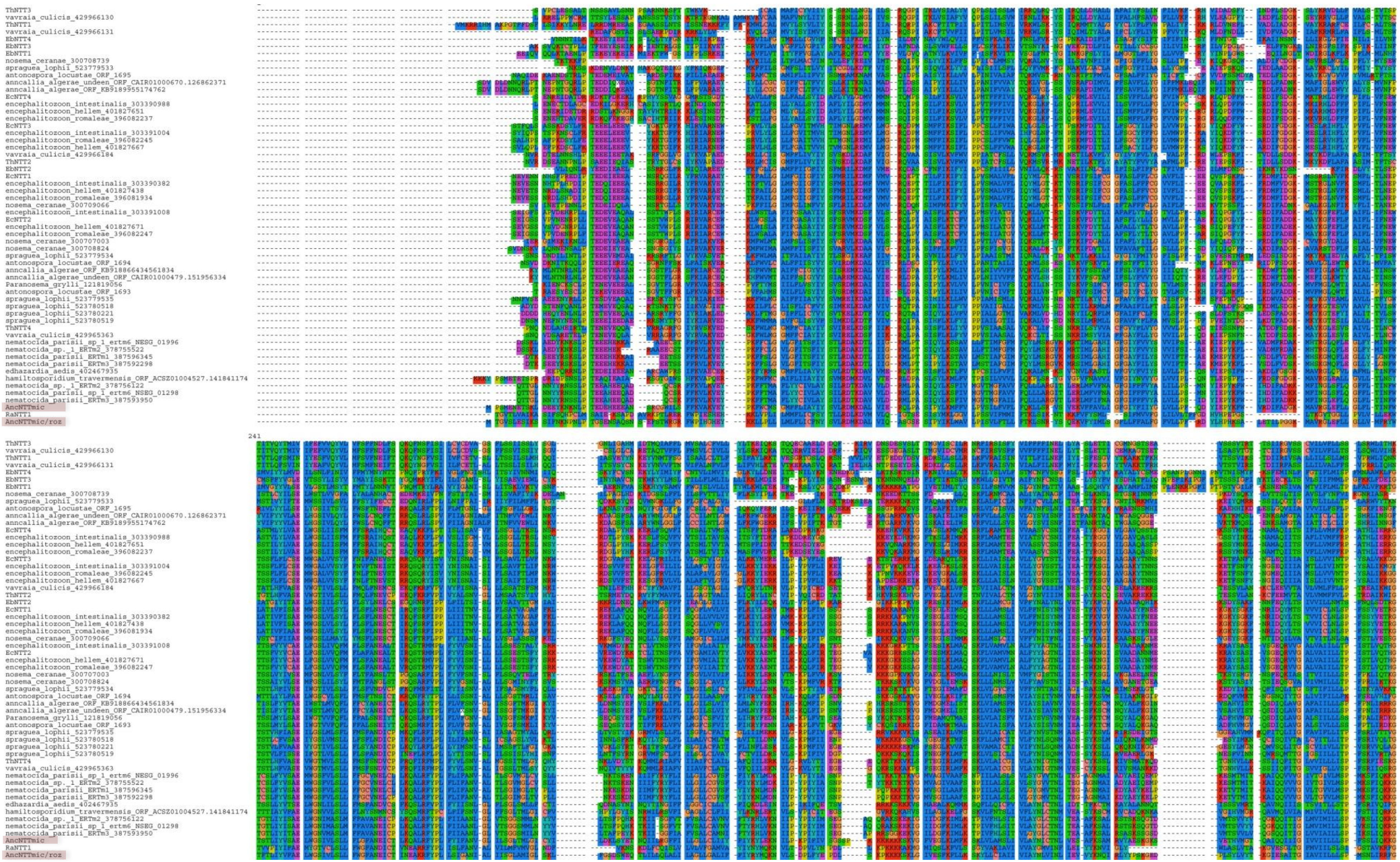

Supplementary Figure 2

Sequence alignment of representative nucleotide transport proteins showing conserved residues across the NTT family including the two ancestral NTT genes (red box). The alignment has been deposited in Figshare (https://doi.org/10.6084/m9.figshare.5170729.v1).

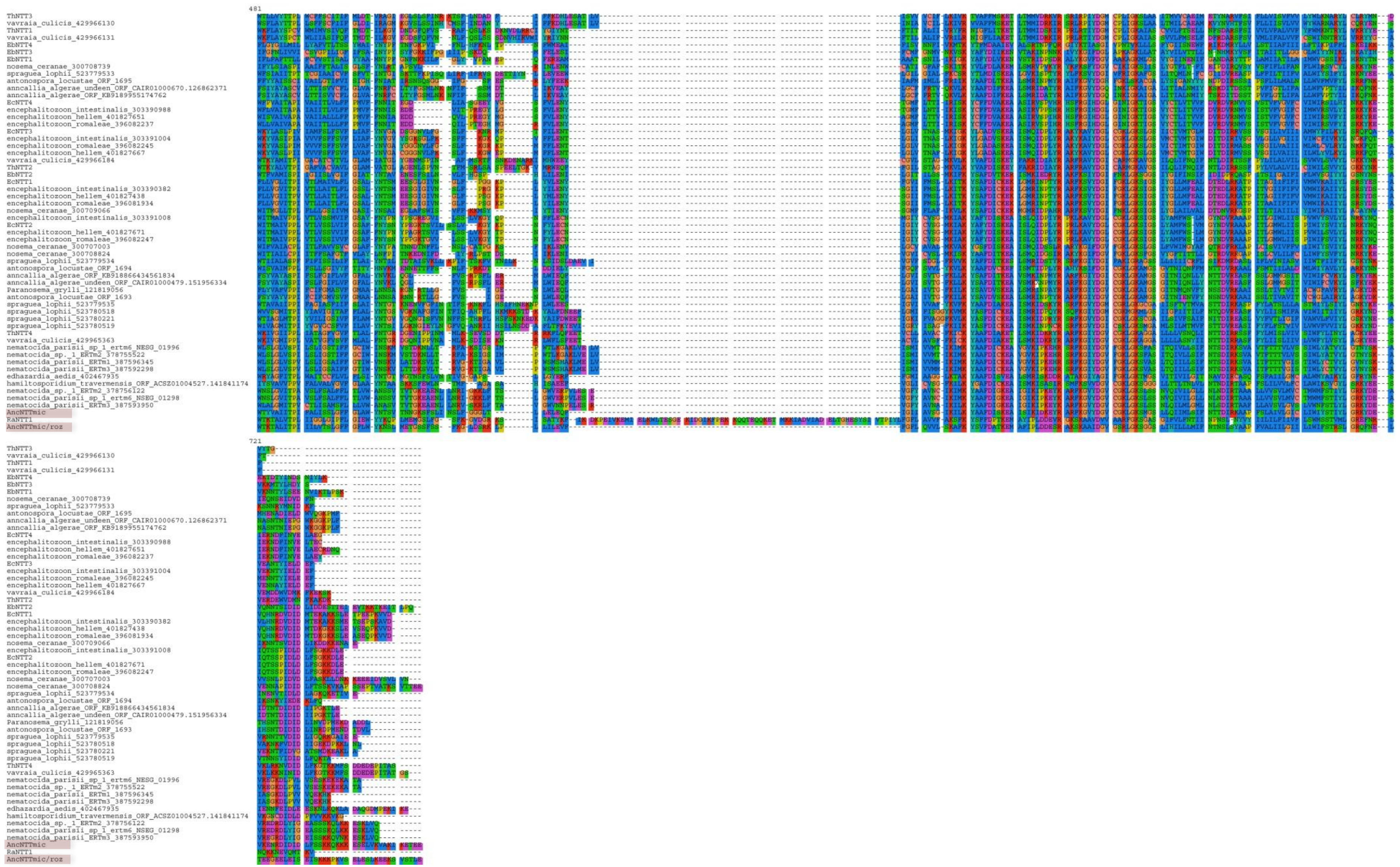

Supplementary Figure 2

Sequence alignment of representative nucleotide transport proteins showing conserved residues across the NTT family including the two ancestral NTT genes (red box). The alignment has been deposited in Figshare (https://doi.org/10.6084/m9.figshare.5170729.v1).

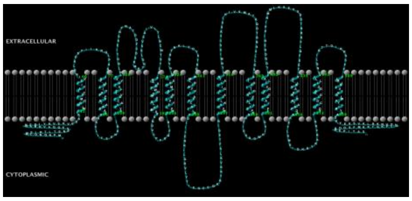

T. hominis ThNTT4

Length: 564
N-terminus: IN
Number of transmembrane helices: 12
Transmembrane helices: 43-60 77-96 109-128 174-192 205-224 241-260 297-316 349-368 381-400 435-454 473-492 509-528

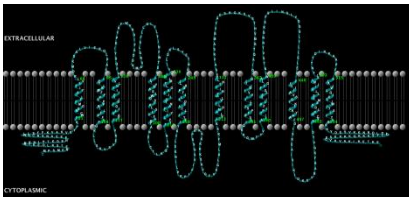

Ancestral $AncNTT_{Mic}$

Length: 575
N-terminus: IN
Number of transmembrane helices: 12
Transmembrane helices: 51-68 85-104 117-136 180-200 213-236 249-268 313-331 362-381 390-409 440-457 482-502 515-534

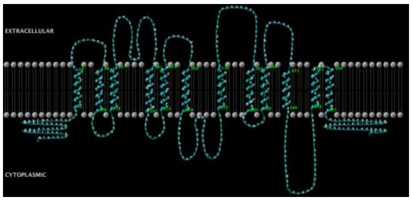

Ancestral $AncNTT_{Roz/Mic}$

Length: 574
N-terminus: IN
Number of transmembrane helices: 12
Transmembrane helices: 52-69 86-105 118-137 181-201 214-233 250-269 312-330 361-380 389-408 431-448 486-503 508-527

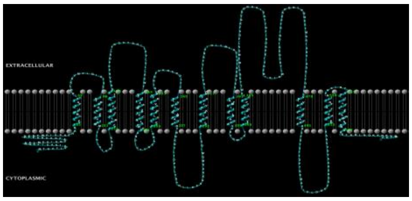

Rozella allomycis RaNTT1

Length: 597
N-terminus: IN
Number of transmembrane helices: 12
Transmembrane helices: 52-69 86-103 116-138 169-191 204-223 240-257 292-311 342-359 368-385 478-495 533-550 557-579

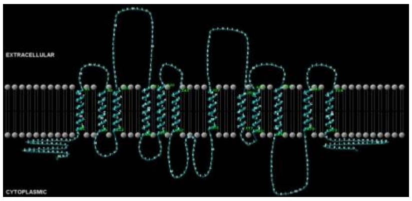

*E. cuniculi Ec*NTT1

Length: 559
N-terminus: IN
Number of transmembrane helices: 12
Transmembrane helices: 46-65 80-99 112-132 171-194 207-230 243-262 305-323 356-373 380-401 416-439 470-489 504-523

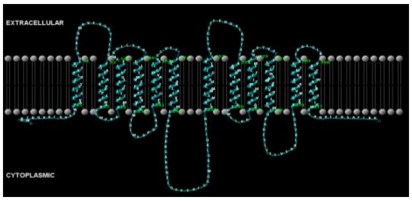

P. amoebophila PamNTT4 (**CAF23209)**

Length: 431
N-terminus: IN
Number of transmembrane helices: 12
Transmembrane helices: 15-34 53-72 79-100 109-132 145-164 171-194 229-252 271-290 297-320 327-349 370-389 398-417

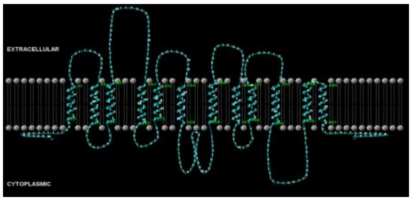

P. amoebophila PamNTT5 (**CAF24067)**

Length: 489
N-terminus: IN
Number of transmembrane helices: 12
Transmembrane helices: 19-35 54-73 86-105 143-162 175-191 210-228 269-288 309-327 340-357 376-395 426-444 449-467

a

b

Apparent K_m (µM) and V_{max} (pmol min^{-1} mg^{-1})							
T. hominis		*E. bieneusi*		*E. cuniculi**		Others	
ThNTT1	7.0 (16.8)	EbNTT1	5.25 (73.5)	EcNTT1	11.4 (3.2)	RaNTT1	0.6 (11.3)
ThNTT2	0.6 (23)	EbNTT2	11.9 (93.0)	EcNTT2	19.8 (11.4)	$AncNTT_{Roz/Mic}$	0.8 (30)
ThNTT3	9.2 (109)	EbNTT3	5.69 (16.8)	EcNTT3	24.2 (8.0)	$AncNTT_{Mic}$	1.9 (28)
ThNTT4	1.2 (38.5)	EbNTT4	0.54 (24.4)	EcNTT4	2.6 (7.2)		

Supplementary Figure 3

(a) Transmembrane topological models of contemporary and ancestral nucleotide transporter proteins. 12 canonical transmembrane domains are shown in each case. Obtained using HMMTOP and represented using TMRPres2D (http://bioinformatics.biol.uoa.gr/TMRPres2D). **(b)** K_m and V_{max} for Microsporidia and ancestral NTTs, determined in this and other studies. *Published values for *E. cuniculi*[14].

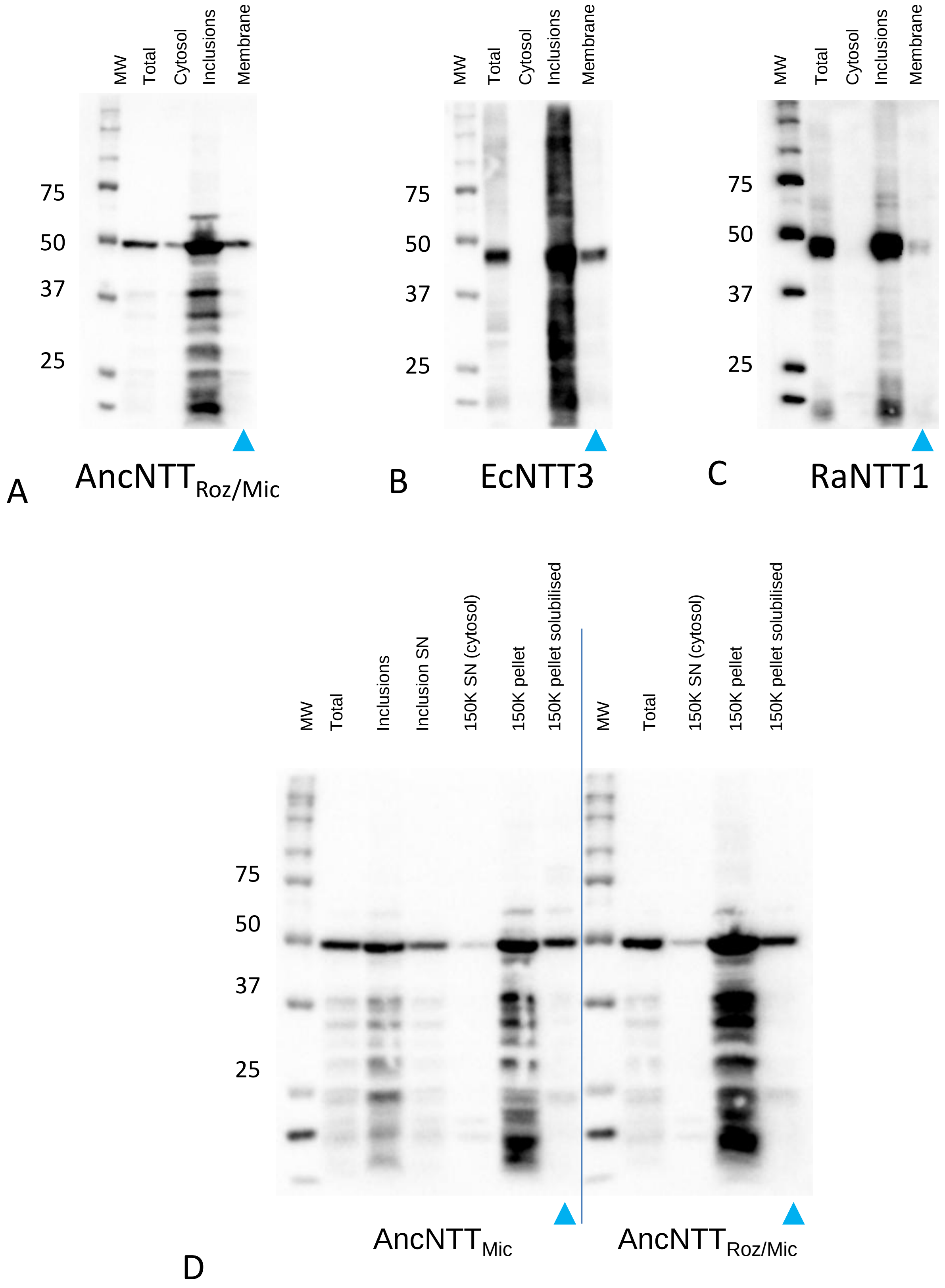

Supplementary Figure 4

Western blot showing membrane fractions of *E. coli* cells expressing ancestral NTTs (**A** and **D**) (AncNTT$_{Roz/Mic}$ and AncNTT$_{Mic}$) and NTTs from two contemporary species (**B** and **C**). Truncated versions of these blots are shown in **Fig. 1c**. NTTs were detected using an anti-HIS antibody. Total = sonicated bacteria, cytosol = 150,000g supernatant, Inclusions = 20,000g pellet, membrane = 150,000g pellet. The membrane fraction is the DDM-solubilised 150,000g pellet (indicated with blue arrow heads).

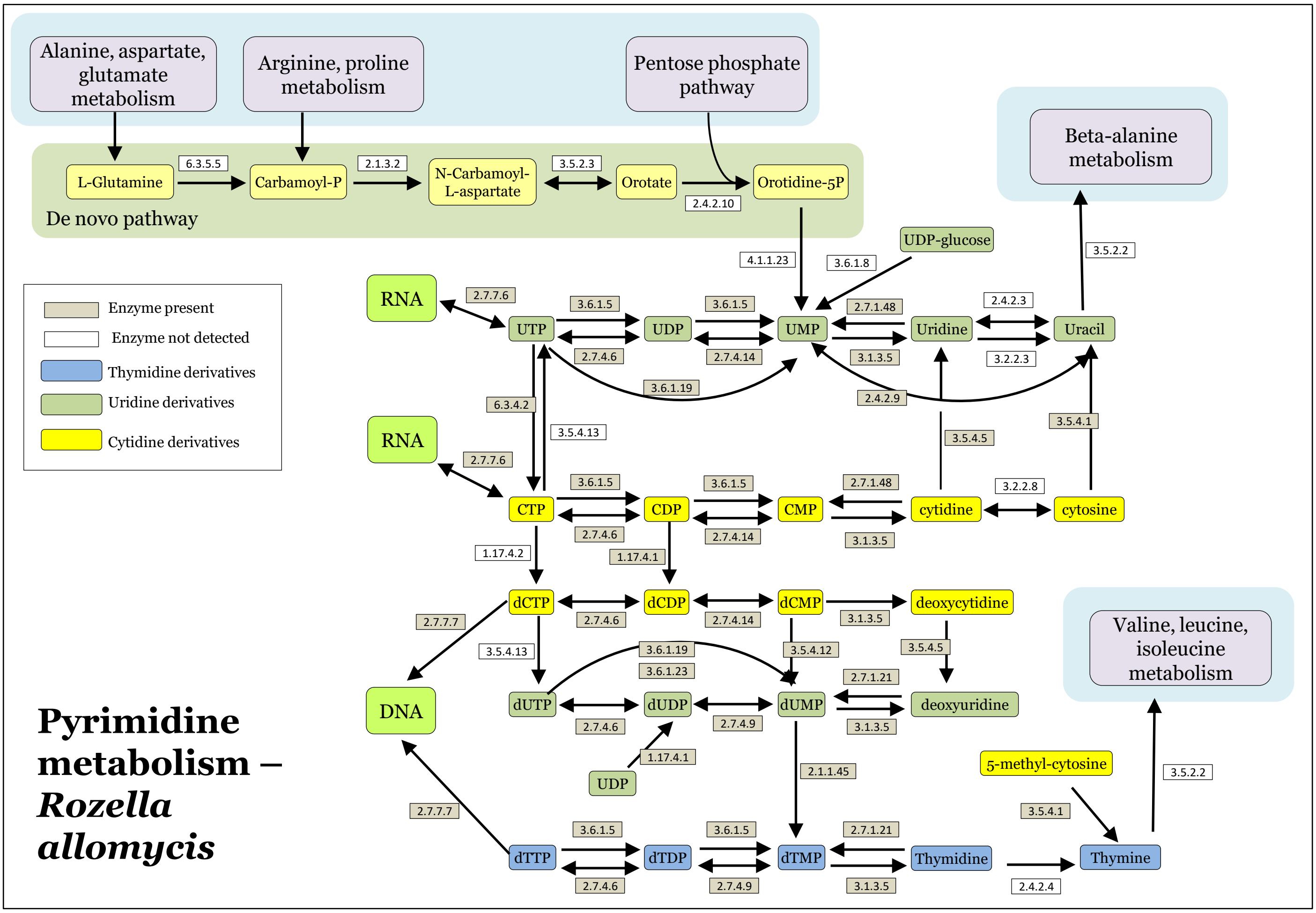

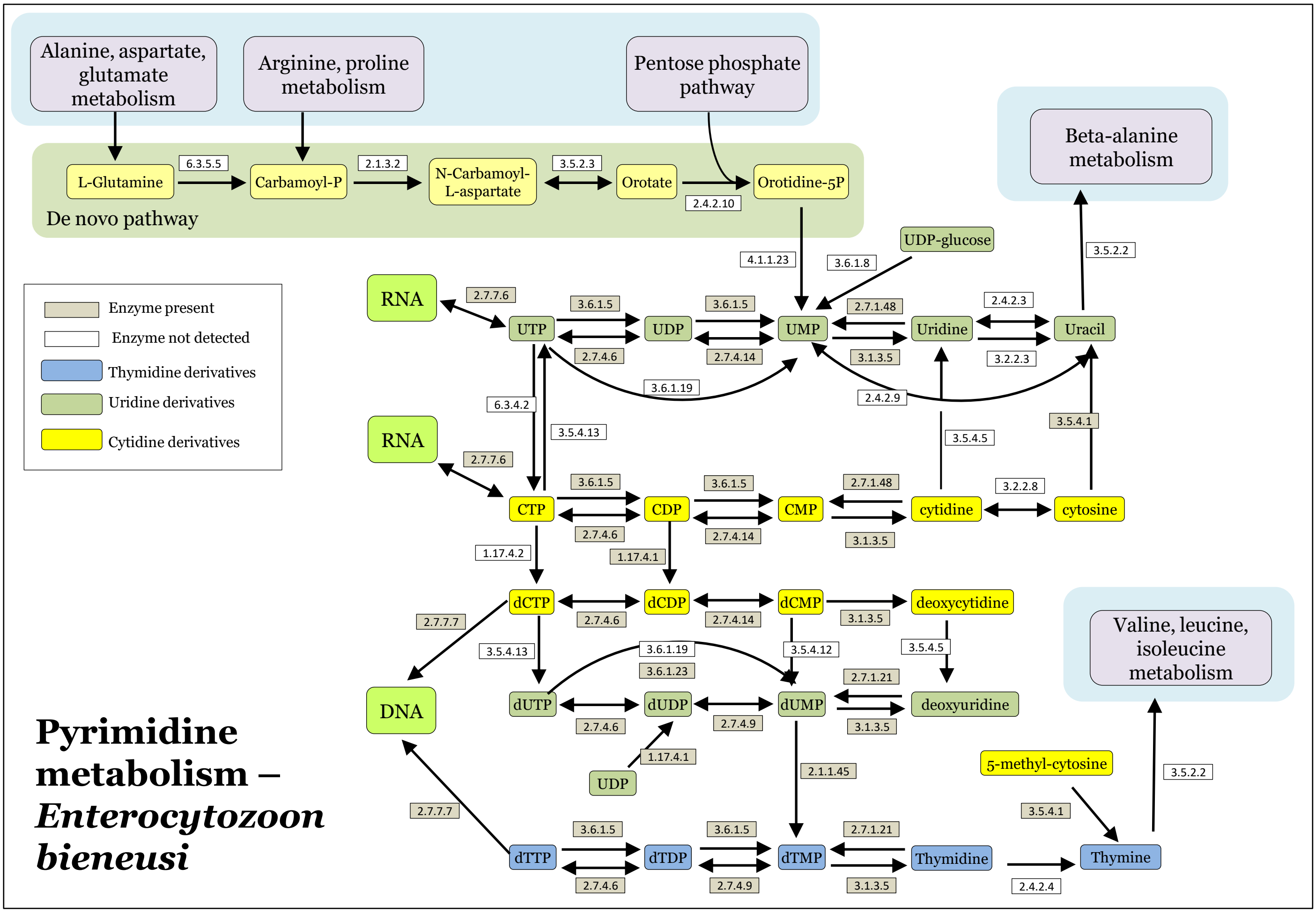

Supplementary Figure 5

Models for the pyrimidine metabolism of *Rozella allomycis* and *Enteroctozoon bieneusi* based upon analyses of their genomes (see Methods section).

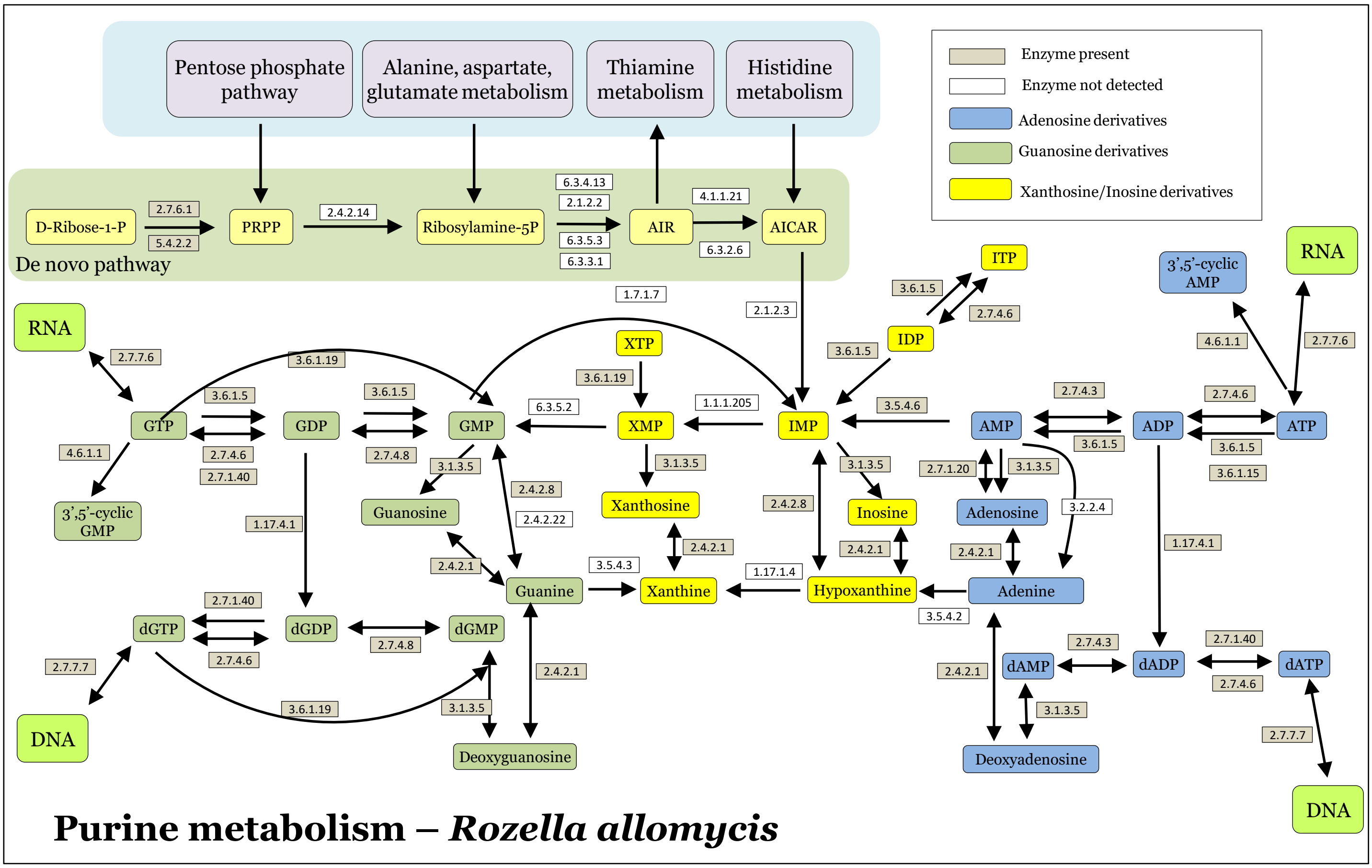

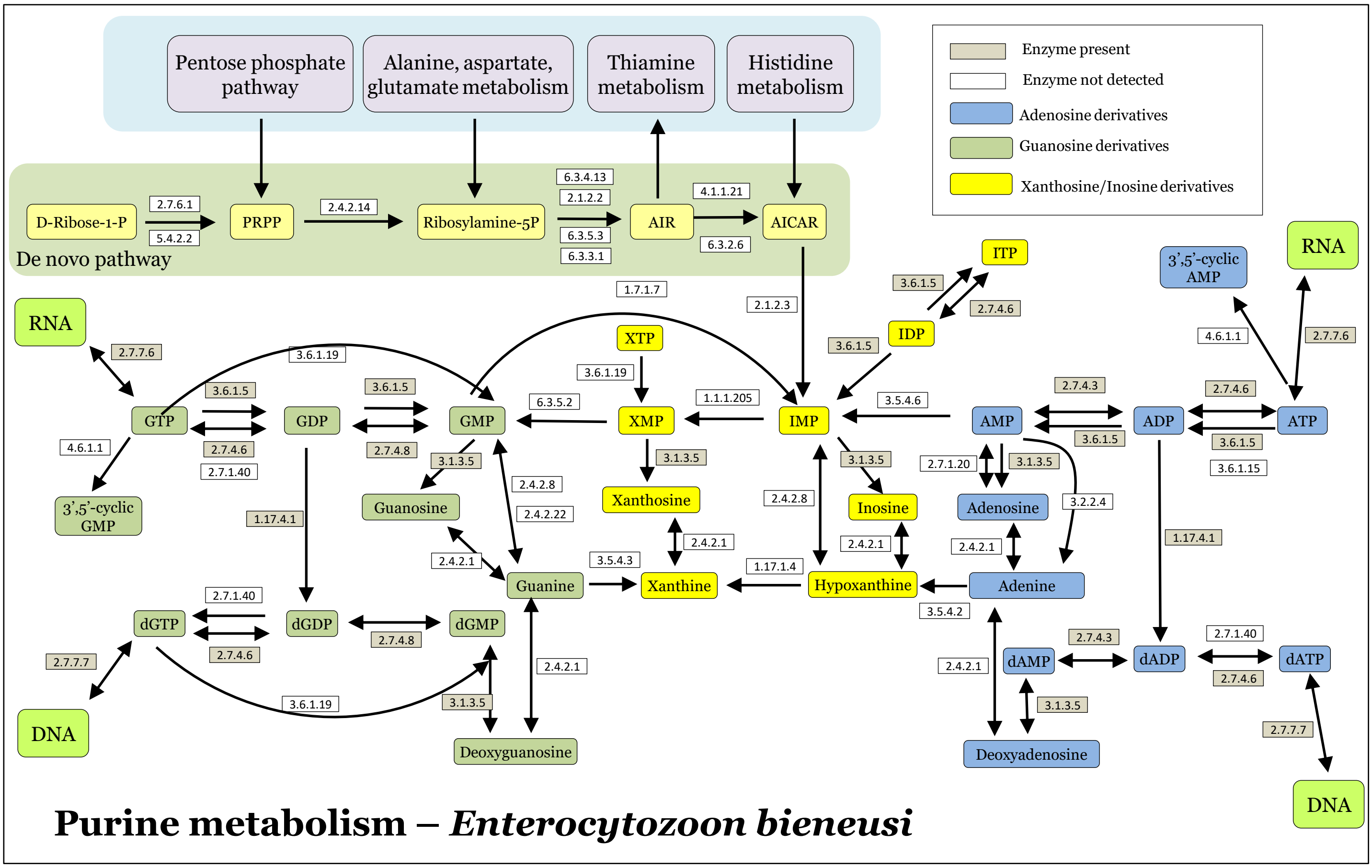

Supplementary Figure 6
Models for the purine metabolism of *Rozella allomycis* and *Enteroctozoon bieneusi* based upon analyses of their genomes (see Methods section).

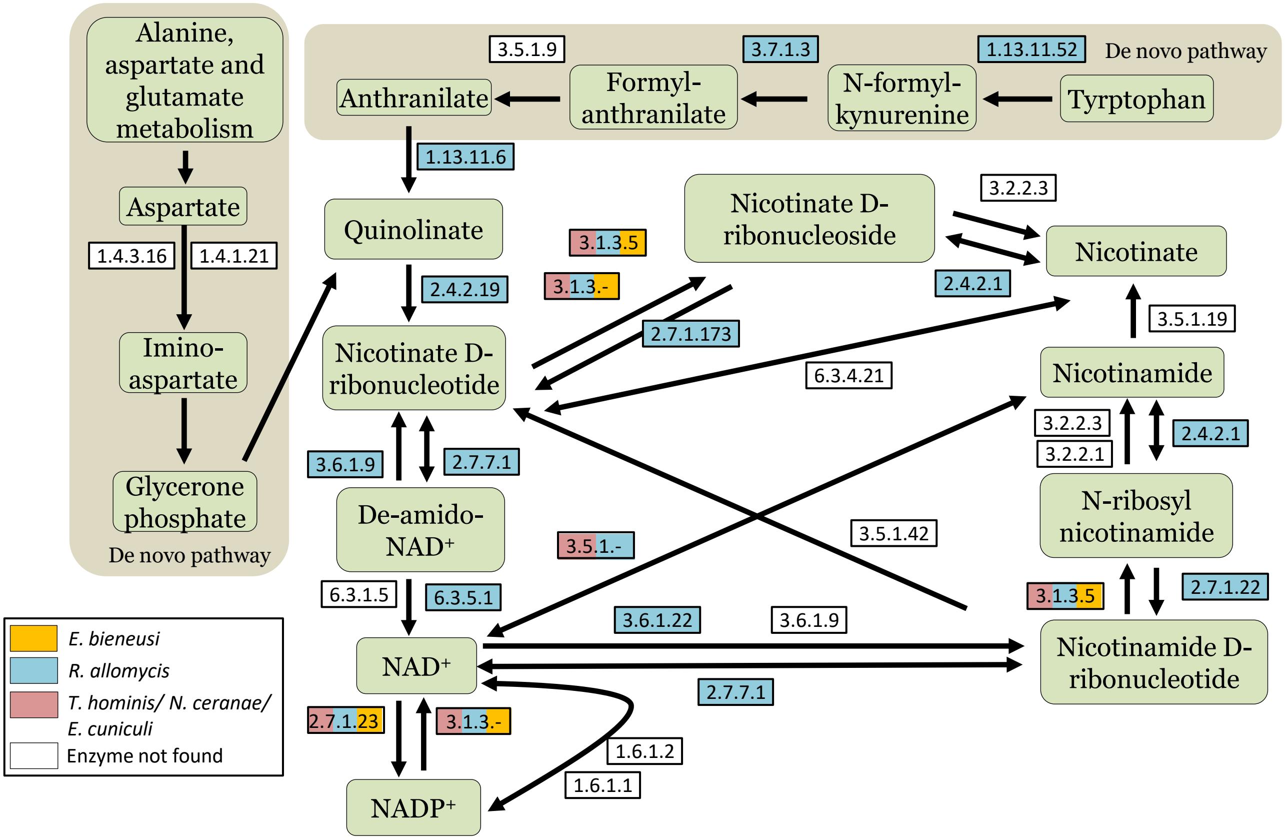

Gene name	EC number	*S. cerevisiae*	*E. cuniculi*	*N. ceranae*	*T. hominis*	*E. bieneusi*	*R. allomycis*
3-hydroxyanthranilate 3,4-dioxygenase	1.13.11.6	YJR025C	-	-	-	-	EPZ36830.1
Kynurenine formidase	3.5.1.9	YDR428C	-	-	-	-	-
Idoleamine 2,3-dioxygenase	1.13.11.52	YJR078W	-	-	-	-	EPZ32120.1
Same as 1.13.11.52	1.13.11.11	-	-	-	-	-	-
Kynureninase	3.7.1.3	YLR231C	-	-	-	-	EPZ32857.1
Nicotinate-nucleotide diphosphorylase	2.4.2.19	YFR047C	-	-	-	-	EPZ36027.1
Aspartate oxidase	1.4.3.16	-	-	-	-	-	-
Aspartate dehydrogenase	1.4.1.21	-	-	-	-	-	-
Nicotinate phosphoribosyltransferase	6.3.4.21	YOR209C	-	-	-	-	-
Nicotinamide-nucleotide adenylyltransferase	2.7.7.1	YGR010W	-	-	-	-	EPZ34376.1
NAD(+) diphosphatase	3.6.1.22	YGL067W	-	-	-	-	EPZ33371.1
Nucleotide diphoshatase	3.6.1.9						-
NAD$^+$ synthase	6.3.5.1	YHR074W	-	-	-	-	EPZ35167.1
NAD-dependent histone deacetylase	3.5.1.-	YDL042C	ECU03_0460	NCER_101498	orf_2231	-	EPZ33665.1, EPZ34088.1
Nicotinamide nucleotide amidase	3.5.1.42	-	-	-	-	-	-
Ribosylnicotinamide kinase	2.7.1.173	YNL129W	-	-	-	-	EPZ33801.1
NAD$^+$ synthase	6.3.1.5	-	-	-	-	-	-
NAD$^+$ kinase	2.7.1.23	YJR049C	ECU07_1050	NCER_100535	orf_1313	-	EPZ34265.1
Pyrimidine and pyridine-specific 5'-nucleotidase	3.1.3.-	YGL224C	ECU05_1170	NCER_102028	orf_2072, orf_1839, orf_1257	EBI_21719	EPZ31299.1
NADP transhydrogenase	1.6.1.1	-	-	-	-	-	-
NADP transhydrogenase	1.6.1.2	-	-	-	-	-	-
5'-nucleotidase	3.1.3.5	-	ECU05_1170	NCER_102028	orf_2072, orf_1839	EBI_21719	EPZ31299.1
Ribosylnicotinamide kinase	2.7.1.22	YNL129W	-	-	-	-	EPZ33801.1
Nicotinic acid riboside hydrolase	3.2.2.3	YDR400W	-	-	-	-	-
Purine nucleosidase	3.2.2.1	-	-	-	-	-	-
Purine nucleoside phosphorylase	2.4.2.1	YLR209C	-	-	-	-	EPZ30827.1
Nicotinamidase	3.5.1.19	YGL037C	-	-	-	-	-

Supplementary Figure 7

NADH biosynthetic pathways. The upper diagram shows the NADH pathway based upon the KEGG database showing the presence or absence of enzymes from Microsporidia and *R. allomycis*. The lower table lists the genes found in the relevant genome sequences.

***Rozella allomycis* RaNTT1**
Ral_1541NdeIF: TGCACCCATATGACCGGGGTCACCCTTGTA
Ral_1541BamHIR:
CACTTGGGATCCTTAAACTTTAGTCATTTG

***E. bieneusi* NTT2**
EBI_21953NdeIF: TGCACCCATATGGTATTAATTCAAAATTTA
EBI_21953BamHIR:
CACTTGGGATCCTTATTGTGGAAGAGTAAT

***E. bieneusi* NTT1**
EBI_24423XhoIF:
TGCACCCTCGAGATGGAAGAAATACAAGTG
EBI_24423BamHIR:
CACTTGGGATCCTTATTTACTTGGTAGGGT

***E. bieneusi* NTT4**
EBI_26694NdeIF: TGCACCCATATGGTAAATAATAATACTATA
EBI_26694BamHIR:
CACTTGGGATCCTTATTTTAAGTAAATATT

***E. bieneusi* NTT3**
EBI_25389XhoIF:
TGCACCCTCGAGATGGCTAAATCAGTACAG
EBI_25389BamHIR:
CACTTGGGATCCTTAACTATAATCGTGTAA

Supplementary Table 1 - Primers used in this study

Rank	Pfam accession	Name of Pfam domain	Probability	E-value	P-value	Score		Aligned columns	Query HMM	Template HMM	% coverage alignment
1	PF03219	TLC TLC ATP/ADP transporter	100	5.70E-23	3.60E-27	198.5		469	6-530	1-491 (491)	96
2	PF05977	MFS_3 Transmembrane secretion effector	100	1.60E-24	1.00E-28	217.4		391	30-536	7-405 (524)	75
3	PF07690	MFS_1 Major Facilitator Superfamily	99.9	6.90E-23	4.40E-27	194.9		343	38-486	1-346 (346)	99
4	PF01306	LacY_symp LacY proton/sugar symporter	99.9	3.50E-21	2.20E-25	187.1		400	29-537	3-412 (413)	97
5	PF03209	PUCC PUCC protein	99.9	2.70E-21	1.70E-25	187.5		371	56-530	2-401 (402)	92
6	PF13347	MFS_2 MFS/sugar transport protein	99.9	1.40E-20	9.10E-25	183.9		384	36-529	2-422 (423)	91
7	PF03825	Nuc_H_symport Nucleoside H+ symporter	99.9	4.20E-20	2.70E-24	179		389	36-529	5-398 (400)	97
8	PF01770	Folate_carrier Reduced folate carrier	99.9	8.30E-20	5.20E-24	174		353	33-486	5-391 (410)	86
9	PF11700	ATG22 Vacuole effluxer Atg22 like	99.9	3.30E-19	2.10E-23	176.5		404	26-536	1-473 (473)	85
10	PF06963	FPN1 Ferroportin1 (FPN1)	99.9	1.30E-18	8.20E-23	168.6		360	33-488	2-411 (432)	83
11	PF00083	Sugar_tr Sugar (and other) transporter	99.9	6.00E-20	3.80E-24	180.9		369	66-537	44-447 (449)	82
12	PF03137	OATP Organic Anion Transporter Polypeptide (OATP) family	99.8	2.50E-18	1.60E-22	172.9		366	35-495	5-558 (583)	63
13	PF06609	TRI12 Fungal trichothecene efflux pump (TRI12)	99.8	3.90E-17	2.50E-21	163.8		385	2-490	13-466 (598)	64
14	PF03092	BT1 BT1 family	99.8	5.80E-18	3.70E-22	164.6		385	45-537	1-416 (425)	91
15	PF05631	DUF791 Protein of unknown function (DUF791)	99.7	1.70E-14	1.00E-18	134.7		303	15-399	20-331 (354)	86

Supplementary Table 2

Top 15 HHPred non-redundant hit list (E-value < 1E-10) for a broad NTT alignment against the Pfam database. All listed profiles are member of the MFS clan CL0015 (http://pfam.xfam.org/clan/CL0015). The search was performed at the HHPred webserver (https://toolkit.tuebingen.mpg.de/hhpred) with the default settings and the database set to “Pfam_04jul16”[47].

Species and sequence identifier (gi number)	Sequence name	Branch lengths		
		Mean	2.50%	97.50%
Enterocytozoon_bieneusi_269861521	EbNTT1	7.627153	7.5951475	7.659159
Enterocytozoon_bieneusi_169806614	EbNTT2	5.10328	5.0798432	5.126716
Enterocytozoon_bieneusi_269859527	EbNTT3	9.107341	9.0685861	9.146096
Enterocytozoon_bieneusi_269861022	EbNTT4	9.694341	9.6510818	9.7376
Encephalitozoon_cuniculi_19173457	EcNTT1	4.333386	4.3124368	4.354335
Encephalitozoon_cuniculi_19074663	EcNTT2	4.519179	4.497648	4.54071
Encephalitozoon_cuniculi_19074661	EcNTT3	5.096813	5.074268	5.119358
Encephalitozoon_cuniculi_19074651	EcNTT4	5.869637	5.8434254	5.895848
Rozella_allomycis_528896761	RaNTT1	1.906874	1.892105	1.921643
Trachipleistophora_hominis_440491551	ThNTT1	8.20875	8.1716995	8.2458
Trachipleistophora_hominis_440493394	ThNTT2	4.70148	4.6781015	4.724858
Trachipleistophora_hominis_440491550	ThNTT3	7.676016	7.6408708	7.711161
Trachipleistophora_hominis_440491582	ThNTT4	4.028978	4.008416	4.049539

Supplementary Table 3

Bayesian relative rates test based upon variation in patristic distances (summed branch lengths) from the common ancestor of Microsporidia and *Rozella* to the duplicated NTT genes in each species. We computed these distances from the branch lengths sampled in our Bayesian phylogenetic analysis by MCMC. The 95% credible intervals for the branch lengths do not overlap, suggesting significant and very substantial variation in evolutionary rate since the common ancestor of the sequences being compared. For example, the NTT genes of *Enterocytozoon bieneusi* have accumulated many more amino acid substitutions than the single NTT gene of *Rozella allomycis* since their split. And *T. hominis* ThNTT4 and ThNTT2 have accumulated many fewer amino substitutions than ThNTT1 and ThNTT3 (see discussion in main text). Distances are given as expected number of amino acid substitutions per site. Values are provided for the mean, 2.75, and 97.5 percentiles of each branch length distribution.

Supplementary Reference

1. Remmert, M., Biegert, A., Hauser, A. & Soding, J. HHblits: lightning-fast iterative protein sequence searching by HMM-HMM alignment. *Nature Meth* **9**, 173-175, doi:10.1038/nmeth.1818 (2011).
