# Supplementary material for: Transporter gene acquisition and innovation in the evolution of Microsporidia intracellular parasites
Source: Nat Commun. 2018 Apr 27;9:1709. doi: 10.1038/s41467-018-03923-4 (PMC5923384; doi:10.1038/s41467-018-03923-4)
Supplement: Supplementary file 2 — Description of Additional Supplementary Files [file 41467_2018_3923_MOESM2_ESM.pdf]

Description of Additional Supplementary Files

File Name: Supplementary Data 1
Description: Amino acid per site posterior probabilities for AncNTTmic/Roz

File Name: Supplementary Data 2
Description: Amino acid per site posterior probabilities for AncNTTmic
